# Stable Colonization of Orally Administered *Lactobacillus casei* SY13 Alters the Gut Microbiota

**DOI:** 10.1155/2020/5281639

**Published:** 2020-02-13

**Authors:** Yuanchun Yue, Xiaoxi Xu, Baoyu Yang, Jing Lu, Shuwen Zhang, Liu Liu, Khaled Nassar, Cai Zhang, Min Zhang, Xiaoyang Pang, Jiaping Lv

**Affiliations:** ^1^Beijing Advanced Innovation Center for Food Nutrition and Human Health, Beijing Technology & Business University (BTBU), Beijing 100048, China; ^2^Institute of Food Science and Technology, Chinese Academy of Agricultural Sciences, Beijing 100193, China; ^3^College of Food Science, Northeast Agricultural University, Harbin 150030, China; ^4^Laboratory of Environment and Livestock Products, Henan University of Science and Technology, Luoyang 471023, China

## Abstract

The gut microbiota plays an important role in intestinal health. Probiotics such as *Lactobacillus* are known to regulate gut microbes and prevent diseases. However, most of them are unable to colonize their stability in hosts' intestinal tracts. In this study, we investigated the ability of *Lactobacillus casei* SY13 (SY13) to colonize the intestinal tract of BALB/c mice, after its oral administration for a short-term (once for a day) and long-term (once daily for 27 days) duration. Furthermore, we also evaluated the influence of its administration on the gut microbial structure and diversity in mice. Male BALB/c mice were gavaged with 10^8^ colony-forming units (CFU) of SY13, and TaqMan-MGB probe and Illumina MiSeq sequencing were performed to assess the colonization ability and bacterial community structure in the cecum contents. The results showed that long-term treatment with SY13 enhanced its ability to form a colony in the intestine tract in contrast to the short-term treatment group, whose colony was retained for only 3 days. Oral administration of SY13 also significantly enhanced the gut microbial diversity. Short-term treatment with SY13 (SSY13) elevated *Firmicutes* and diminished *Bacteroidetes* phyla compared with long-term treatment (LSY13) and controls. The findings laid the foundation for the study of probiotic colonization ability and improvement of microbiota for the prevention of gut diseases.

## 1. Introduction

The gut microbiota is a complex microecosystem in the intestinal tract that includes numerous bacteria [1–3]. The balance, composition, and diversity of the gut microbiota are closely correlated with host metabolism, energy regulation, immune development, pathogen defense, and prevention of intestinal diseases such as inflammatory bowel disease (IBD) and colorectal cancer [2]. However, this homeostasis is implemented by intricate interactions between the microbiome and the host. Therefore, prevention of diseases by gut microbes has attracted much research attention, with particular focus on microbial composition and diversity.

Lactic acid bacteria (LAB) probiotics and some of their secreted bioactive components can prove beneficial for intestinal tract health by influencing microbial diversity [4–7]. Several LAB including *Lactobacillus casei* BL23, *L*. *casei* Zhang, and *L*. *rhamnosus* GG are reported to prevent colitis and/or colon cancer when applied orally, and all improve the gut microbiota [8–10]. Probiotics can promote microbial richness and bacterial diversity in the gut microbiota of model mice with an imbalanced gut microbiota [11]. Borrelli et al. had evidence that probiotic could modulate the microbiota of gut and brain axis and behaviour in zebrafish [12]. And Lorena explored the function of probiotic and gut microbiota by proteomics [13]. Furthermore, Xin et al. proved that *L. johnsonii* BS15 could modulate immunity of intestinal and intestinal microbiota in piglets [14]. Moreover, LAB is reported to prevent allergies, control blood cholesterol levels, and regulate immune function by influencing gut microbial development [15–17].

We previously reported that *L. casei* SY13, isolated from fermented dairy products, can improve intestinal diseases and help to maintain host health [18]. Moreover, we found that *L. casei* SY13 reduced fat deposition in Syrian golden hamsters in a time- and dose-dependent manner [19]. However, the colonization ability of *L. casei* SY13 and the effects of oral administration its bacteria on gut microbiota alteration remain unclear. Therefore, the aim of the present study was to evaluate the colonization ability of *L. casei* SY13 and explore its effects on gut microbial structure and diversity in mice treated by gavage once (short-term) and 27 times (long-term). The results demonstrate that the stable colonization of *L. casei* SY13 is associated with dosage and lays a foundation for studying interactions between *L. casei* SY13 and other members of the gut microbiota.

## 2. Materials and Methods

### 2.1. Bacterial Culture and Harvesting

*L. casei* SY13 cells used in this study were maintained at −80°C and cultured in 100 mL/250 mL MRS broth medium (CM187, Beijing Luqiao Company, China) at 37°C for 24 h. Cells were harvested by centrifugation at 6010 ×*g* for 4 min, washed three times with phosphate-buffered saline (PBS), and adjusted to 10^9^ colony-forming units (CFU) per mL for oral treatment of mice.

### 2.2. Animals and Experimental Design

All experiments were approved by the Laboratory Animal Welfare and Animal Experimental Ethical Committee of China Agricultural University (approval number: CAU20161020-3). 6- to 8-week-old male BALB/c mice were obtained from Vital River Laboratory Animal Technology Co., Ltd. (Beijing, China). All mice were fed using the laboratory animal management platform (SPF) at China Agricultural University.

Mice were bred as described previously by Jia et al. [19]. Briefly, mice were separated two per cage and reared under a 12 h light/dark cycle with carefully controlled temperature and moisture. Mice were free to eat and drink, and feed and water were sterilized. After 7 days of adaptation to the new environment, mice were divided into three groups: a control group administered PBS and two experimental groups that the mice were treated with *L. casei* SY13 at a short-term for only one day, while long-term treatment was done for 27 days with once-daily inoculations.

### 2.3. Microbial DNA Extraction

The whole cecal tissue from each mouse was collected and stored at −80°C. Then total DNA was extracted using a TIANamp Stool DNA Kit (DP320, Tiangen Company, China) according to the manufacturer's instructions. DNA suspensions were stored at −80°C.

### 2.4. Detection of *L. casei* SY13 in the Intestinal Tract

Real-time PCR (RT-PCR) was carried out at 1, 3, 5, and 7 days after gavage with *L. casei* SY13. The number of bacterial cells in the cecum was calculated, and the retention time of the bacteria in the intestinal tract under short- and long-term treatment was determined. The method of TaqMan-MGB probe (FAM-CTCAAAAATGGATCTTG-MGB) with RT-PCR was used to assess the colonization ability of *L. casei* SY13 according to the previous report of Jia et al. [19]. In briefly, RT-PCR experiments (20 *μ*L) contained 1 *μ*L 06232F (TCAACCGTGACTGGCAAGT, 10 *μ*mol/L), 1 *μ*L 06232R (AGCGGCTTGTCGAACTGA, 10 *μ*mol/L), 1 *μ*L 06232P (10 *μ*mol/L), 1 *μ*L template DNA, 10 *μ*L original TaqMan R Universal PCR Master Mix, and 6 *μ*L sterilized water. The RT-PCR procedure included a 50 min proenzyme activation step, followed by 95°C predenaturation for 10 min, and 60 cycles of denaturation at 95°C for 15 s and annealing and extension at 58°C for 60 s. Fluorescence detection was performed during annealing and extension stages using an ABI7500 instrument (Thermo Fisher Company, Singapore).

### 2.5. 16S rDNA Amplification and High-Throughput Sequencing

The V3-V4 hypervariable region of bacterial 16S rDNA was amplified with primers 338F (5′-ACTCCTACGGGAGGCAGCAG-3′) and 806R (5′-GGACTACHVGGGTWTCTAAT-3′). A 30 ng sample of genomic DNA was added to the 50 *μ*L reaction mixture, and amplification products were separated by 2% agarose gel electrophoresis using an AxyPrep DNA Gel Extraction Kit (Tiangen). The Illumina MiSeq protocol (Allwegene Technology Co. Ltd., Beijing, China) was performed for analyzing the microbial structure and diversity in each group.

### 2.6. Sequence Analysis and Quality Control

High-throughput sequencing was performed using an Illumina MiSeq Sequencer (PE250). Firstly, the obtained FASTQ data were filtered and processed to obtain high-quality sequences, and paired sequences were merged into single sequences based on sequence coverage using FLASH software [20]. Operational taxonomic unit (OTU) information for each group was analyzed and classified at the 97% similarity level. Chao1 and observed species indices were used to assess the richness and diversity of gut microbial communities at the genus level, QIIME v.1.8 was employed for data analysis, and *R* software was used to generate Venn diagrams, bar plots, and heatmaps and to perform principal coordinate analysis (PCoA).

### 2.7. Statistical Analysis

Statistical analysis was carried out using Prism software and displayed as mean ± standard deviation (SD). Differences between two groups were analyzed by Student's *t*-tests, and *p* < 0.05 was considered significant.

## 3. Results and Discussion

### 3.1. *L. casei* SY13 Retention Time

**Figure 1:**
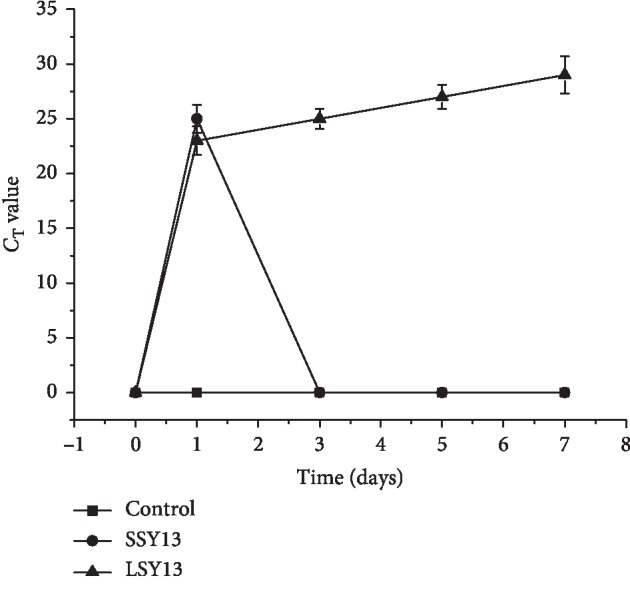
Retention time of *L. casei* SY13 in the cecum of mice. PBS = negative control; SSY13 = a single oral dose of SY13 (short-term treatment); LSY13 = 27 oral doses of SY13 (long-term treatment). Error bars represent means ± SD from three independent experiments.

After short-term oral administration of *L. casei* SY13, cells were maintained in the intestinal tract for <3 days (Figure 1). However, long-term treatment resulted in cells being retained for 7 days, indicating that long-term oral administration of *L. casei* SY13 extended the retention time of this bacterium. Notably, *L. casei* SY13 did not appear in the control group throughout the experimental period.

For LAB to successfully colonize the intestinal tract of LAB, gastric acid and bile salts must be tolerated, and bacterial adhesion must take place [21–23]. Therefore, the colonization ability of LAB in the intestinal tract is very important in evaluating its function. Chen et al. have concluded that the ability of gastrointestinal tract tolerance and bile salts tolerated would influence the selection of *Lactobacillus* [21]. In the previous work, we used TaqMan-MGB RT-PCR, designed primers and probes for the detection of *L. casei* in the intestinal tract of mice, and tested probe specificity, and the previous report has demonstrated the feasibility of this approach [24]. In the present study, we analyzed *L. casei* SY13 in the cecum of mice and found that a longer gavages period enhanced colonization of the intestine. This may contribute to the long-term invasion of SY13 in the intestinal tract and suggests that the intestinal microenvironment encourages the growth of *L. casei* SY13. Extending the gavage period increased the retention time of SY13 in the intestinal tract from 3 days to at least 7 days. However, SY13 did not remain permanently in the intestinal tract, consistent with the fact that LAB is known to affect colonization in the intestine [25].

### 3.2. Bacterial Diversity of the Gut Microbiota

**Figure 2:**
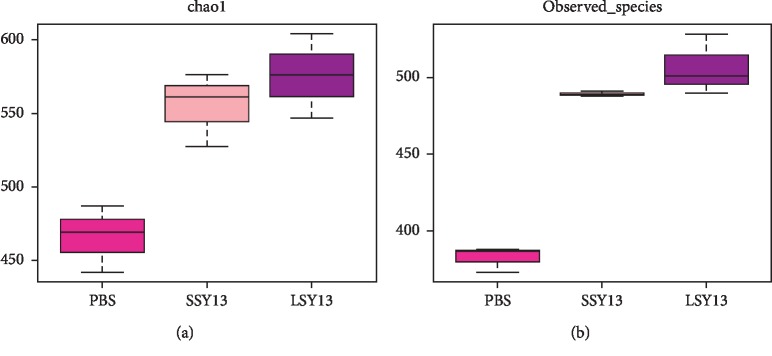
Alpha diversity index of gut microbial.s(a) Chao1 index. (b) Observed species index. PBS = negative control; SSY13 = a single oral dose of SY13 (short-term treatment); LSY13 = 27 oral doses of SY13 (long-term treatment). Error bars represent means ± SD from three independent experiments.

To investigate the functional role of SY13 in determining the richness and diversity of gut microbial communities, we first analyzed the alpha diversity index. Chao1 and observed species indices were significantly elevated (*p* < 0.05) in both SSY13 and LSY13 groups compared with controls, especially in the LSY13 group (Figures 2(a) and 2(b)), indicating that *L. casei* SY13 may increase bacterial richness and diversity in the gut microbiota. The supporting results had been shown by Gao et al. that long-term gavage with probiotics could alter gut microbiota [26]. Moreover, Zhang et al. proved that administration of *Lactobacillus* could change the diversity and composition of gut microbiota in weaned piglets [27]. On the other hand, some researchers studied the ability of probiotic strains to modulate the gut microbiota in mule ducks and found that probiotic treatment had no effect on gut microbial richness and diversity in either ileal and cecal samples initially, but at the end of the overfeeding period, both diversity and richness were decreased following probiotic treatment [28]. However, in the present study, addition of *L. casei* SY13 enhanced both parameters at both time points. The effects of probiotics on the richness and diversity of the gut microbiota differed between mice and mule ducks, presumably due to differences in dietary mode and methods for probiotic treatment. Previous reports showed that increasing species richness and diversity can help to prevent chronic diseases such as asthma, obesity, and inflammatory bowel disease (IBD) [29–31]. In the present study, SY13 appeared to increase these parameters and may therefore relieve diseases caused by diminished microbial diversity.

**Figure 3:**
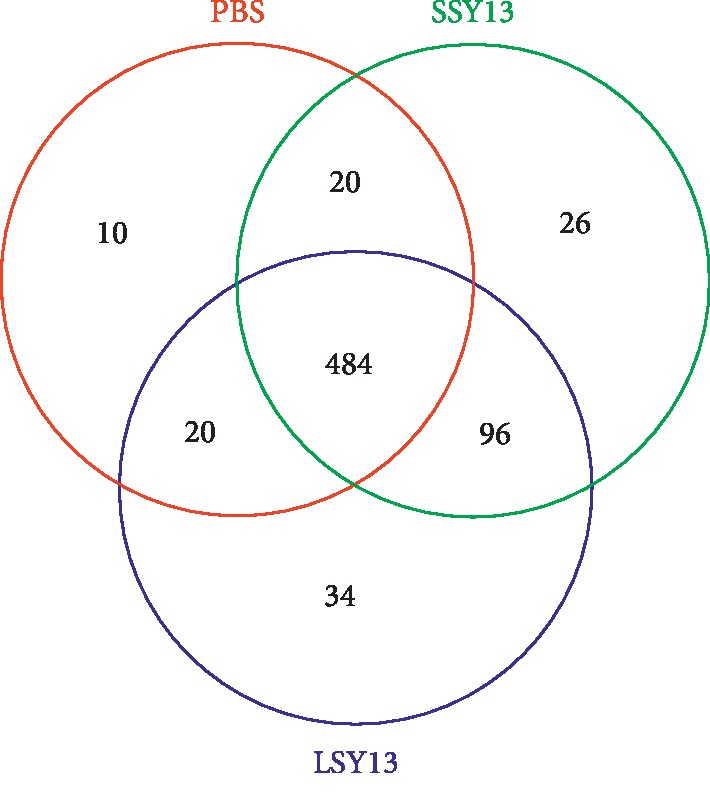
Venn diagram of OTUs. PBS = negative control; SSY13 = a single oral dose of SY13 (short-term treatment); LSY13 = 27 oral doses of SY13 (long-term treatment). Error bars represent means ± SD from three independent experiments.

A Venn diagram was plotted to analyze the similarity and characteristics of OTUs in different samples [21]. We selected OTU samples sharing 97% similarity to statistically analyze the effects of short- and long-term administration of SY13 on gut microbes using different colors for each group (Figure 3). There were 484 common OTUs among PBS (control), SSY13, and LSY13 groups, with 10, 20, and 36 specific OTUs and 534, 626, and 634 total OTUs, respectively. These results indicate that administration of *L. casei* SY13 elevated the diversity of the gut microbiota, consistent with the alpha diversity index results described above (Figure 2).

According to the above results, addition of *L. casei* SY13 appeared to enhance both the diversity and richness of the gut microbiota. However, long-term treatment caused a smaller increase, indicating adaptive responses of intestinal flora to *L. casei* SY13.

### 3.3. Species Composition and Community Structure of the Gut Microbiota

**Figure 4:**
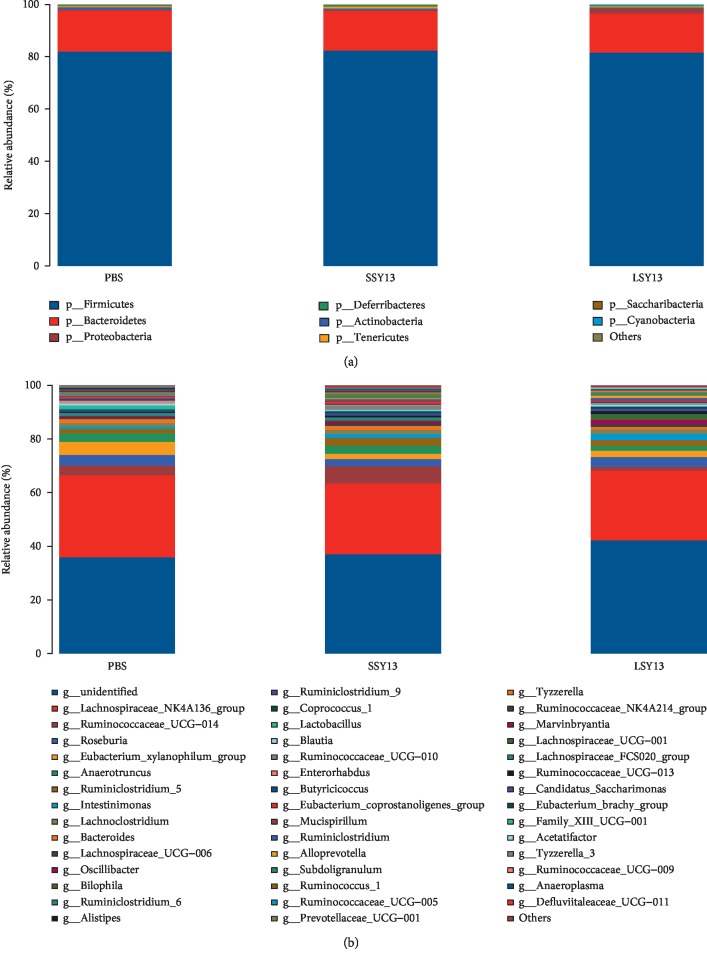
Relative abundance of species in the gut microbiota at phylum (a) and genus (b) levels. PBS = negative control; SSY13 = a single oral dose of SY13 (short-term treatment); LSY13 = 27 oral doses of SY13 (long-term treatment). Error bars represent means ± SD from three independent experiments.

To investigate the effect of short- and long-term oral administration of *L. casei* SY13 on the community structure of the gut microbiota in mice, we tested the relative bacterial abundance in each treatment group. As shown in Figure 4, there were 8 different bacterial phyla (a) and 44 different genera (b). At the phylum level, *Firmicutes* was most abundant in all groups, followed by *Bacteroidetes*. Notably, short-term addition of SY13 enhanced the abundance of the dominant *Firmicutes* compared with control and LSY13 groups (average abundance = 81.95%, 82.35%, and 81.57% for PBS, SSY13, and LSY13, respectively). This may explain the remarkable increase in the *Ruminococcaceae* genus in the SSY13 group and the decrease in this genus in the LSY13 group. Meanwhile, SY13 inhibited the *Bacteroides* phylum compared with the untreated group (15.76%), and short-term addition of SY13 had a greater inhibitory activity (15.0%) than long-term treatment (15.13%). Previous reports showed that *Bacteroides* can promote fat accumulation, which may be related to the inhibition of fasting-induced adipocytokines (FIACS), and elevation of FIACS can inhibit the activity of lipoprotein lipase (LPL), which can reduce fat accumulation and promote fat consumption [32]. Our results indicate that short-term SY13 treatment may be used to manipulate *Bacteroides* in the gut microbiota.

**Figure 5:**
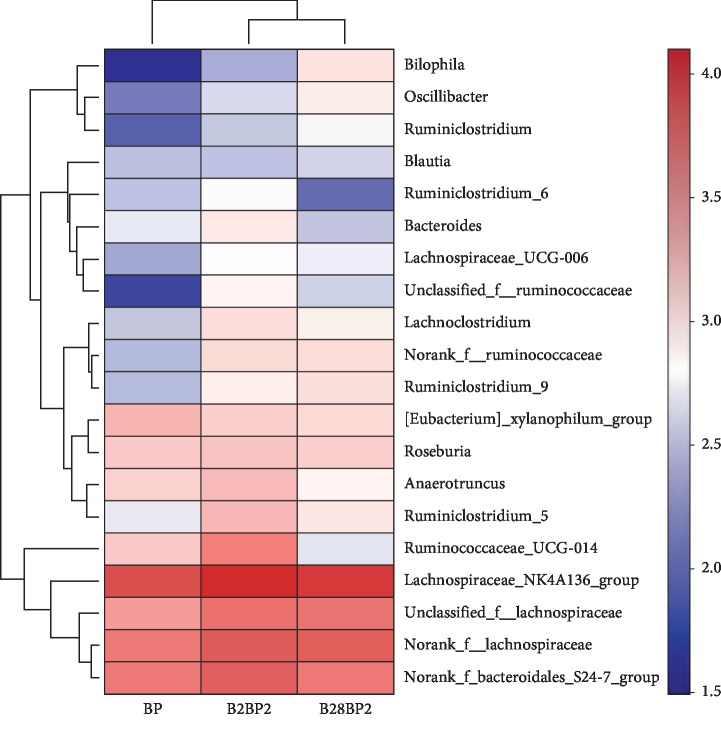
Heatmap of abundant species in the bacterial community at the phylum level. PBS = negative control; SSY13 = a single oral dose of SY13 (short-term treatment); LSY13 = 27 oral doses of SY13 (long-term treatment). Error bars represent means ± SD from three independent experiments.

In order to investigate similarities and differences in species composition in different samples, a heatmap was plotted. The intestinal microbiota in each group is shown at the genus level in Figure 5, based on the top 20 bacterial genera. The abundance of *Bilophila*, *Oscillibacter*, and *Ruminococcaceae* genera was increased significantly with increasing SY13 treatment duration, compared with controls. Clustering analysis classified species and samples into three and two categories, respectively, with short- and long-term addition of SY13 clustered together, consistent with the results of gut microbial diversity, further indicating adaptation of the gut microbes to SY13.

### 3.4. Comparative Analysis of Samples

**Figure 6:**
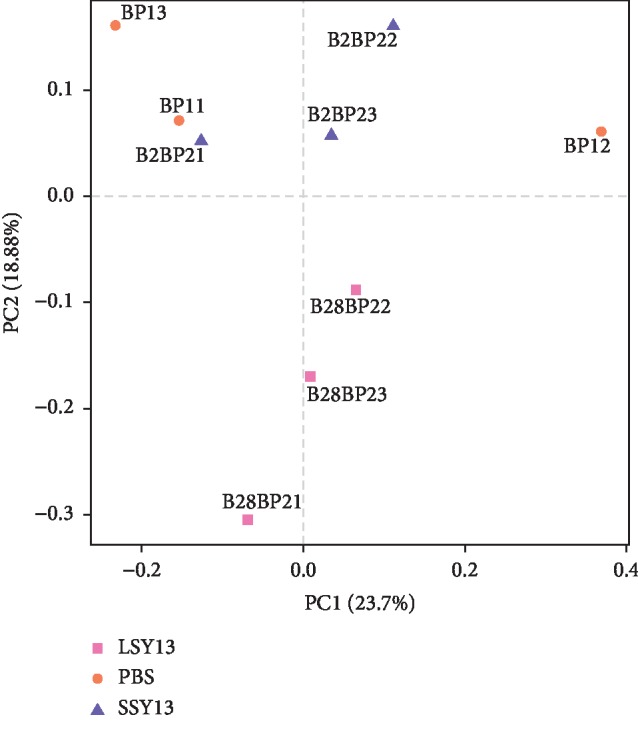
PCoA of OTUs in the mouse gut microbiota. PBS (BP11, BP12, and BP13) = negative controls; SSY13 (B2BP21, B2BP22, and B2BP23) = a single oral dose of SY13 (short-term treatment); LSY13 (B28BP21, B28BP22, and B28BP23) = 27 oral doses of SY13 (long-term treatment).

To assess similarities and differences in the community composition of different samples, beta diversity was explored using PCoA. As shown in Figure 6, three groups were divided into three different regions based on PC1 and PC2, indicating differences in the community composition of the gut microbiota in these groups. The LSY13 group was clearly distinct from the other groups. The results indicate that long-term oral SY13 could drastically transfer gut microbiota when compared with one time oral, which were similar to the results of retention time for SY13. Furthermore, SY13 is a typical LAB, which could produce lactic acid, bacteriocins, and other bioactive compounds to influence the community composition of gut microbial, and long-term oral SY13 contribute to the accumulation of these metabolites in the intestinal tract.

## 4. Conclusion

In this study, we showed that short-term and long-term inoculation with *L. Casei* sy13 can alter the diversity and community structure of intestinal flora in mice. Long-term oral of *L. Casei* sy13 could enhance the ability of colonization in the intestinal tract: however, a single time of oral sy13 had a greater effect on gut microbiota structure at phylum and genus levels than long-term treatment. This may contribute to the environmental adaptation of gut microbiota. These findings may be of relevance for improving the gut microbiota and preventing intestinal tract diseases and give a strategy for lactic acid bacteria to stably colonize the intestinal tract of host.

## Data Availability

The original data are shown in the supplementary material.

## Supplementary Materials

Supplementary Materials The data of OTU.
