# Supplementary material for: Stable Colonization of Orally Administered Lactobacillus casei SY13 Alters the Gut Microbiota
Source: Biomed Res Int. 2020 Feb 13;2020:5281639. doi: 10.1155/2020/5281639 (PMC7040389; doi:10.1155/2020/5281639)
Supplement: Supplementary Materials — The data of OTU. [file 5281639.f1.pdf]

ID	BP11	BP12	BP13	B2BP21	B2BP22	B2BP23	B28BP21
OTU_1	11	53	3	95	76	75	540
OTU_10	20	27	0	33	1	0	0
OTU_100	0	0	0	1	1	3	0
OTU_101	0	2	0	2	3	0	1
OTU_102	0	0	2	0	0	0	0
OTU_103	1	2	0	2	2	3	1
OTU_104	0	1	0	0	1	1	1
OTU_105	0	0	0	2	0	3	0
OTU_106	0	1	1	2	3	0	2
OTU_107	0	1	0	2	1	2	0
OTU_108	1	0	2	0	7	5	2
OTU_109	0	3	0	2	5	2	0
OTU_11	8	6	3	12	10	12	5
OTU_110	0	0	0	1	2	5	1
OTU_111	0	0	0	4	1	0	3
OTU_112	0	0	0	0	1	5	0
OTU_113	0	3	1	1	0	0	5
OTU_114	0	0	0	0	0	0	1
OTU_115	0	1	6	0	6	0	2
OTU_117	1	0	0	0	2	0	1
OTU_118	0	0	0	0	1	0	3
OTU_119	1	0	0	0	1	0	0
OTU_12	5	0	0	14	13	16	3
OTU_121	0	0	0	0	0	0	2
OTU_122	0	1	1	0	3	1	1
OTU_123	0	0	0	0	2	4	1
OTU_124	0	0	0	0	0	0	1
OTU_125	259	55	85	249	139	138	185
OTU_126	0	0	5	1	3	0	3
OTU_127	0	0	1	1	0	1	1
OTU_128	0	0	0	0	1	0	0
OTU_129	0	0	0	0	0	1	1
OTU_13	15	4	5	14	7	4	12
OTU_130	0	0	0	0	2	0	0
OTU_131	0	2	1	1	1	0	0
OTU_132	0	0	0	0	0	2	0
OTU_133	0	0	0	0	0	0	1
OTU_134	0	0	0	1	4	0	0
OTU_135	0	0	0	1	1	0	0
OTU_136	0	0	0	1	0	0	0
OTU_137	0	0	0	1	0	0	0
OTU_138	0	1	1	2	9	1	0
OTU_139	45	37	18	45	47	53	25
OTU_14	7	2	13	11	3	7	18
OTU_140	0	1	0	0	4	1	1
OTU_141	0	0	0	0	0	0	0
OTU_142	429	551	179	284	288	234	214
OTU_143	386	379	164	178	196	165	83
OTU_144	248	84	72	58	55	27	149
OTU_145	30	73	13	9	4	27	6

OTU_146	7	19	18	19	8	11	5
OTU_147	5	31	5	25	15	3	7
OTU_148	0	6	1	0	3	4	2
OTU_149	0	0	3	1	8	1	2
OTU_15	2	0	0	2	14	7	0
OTU_150	0	0	0	0	5	2	0
OTU_151	0	0	0	2	1	3	2
OTU_152	1	2	0	0	3	0	2
OTU_153	0	1	0	1	9	1	1
OTU_154	0	0	0	1	3	1	2
OTU_155	0	0	0	1	0	0	2
OTU_156	1	2	1	2	2	0	1
OTU_157	0	7	0	1	2	3	0
OTU_158	0	0	0	0	5	0	3
OTU_159	0	1	0	1	6	1	4
OTU_16	0	6	6	5	36	0	0
OTU_160	0	3	4	1	8	1	8
OTU_161	0	0	1	0	4	1	0
OTU_162	0	6	0	3	2	3	3
OTU_163	0	0	0	0	0	0	0
OTU_164	1	2	7	1	11	2	5
OTU_165	0	1	0	1	0	1	4
OTU_166	0	2	0	1	0	1	1
OTU_167	0	0	0	0	0	1	0
OTU_168	1	1	1	5	6	5	1
OTU_169	0	0	0	1	0	1	3
OTU_17	1	1	0	6	11	0	1
OTU_170	0	0	0	0	0	2	3
OTU_171	0	1	0	2	1	2	0
OTU_172	0	0	0	0	2	1	0
OTU_173	0	1	0	1	1	0	3
OTU_174	0	1	0	2	1	0	0
OTU_175	1	3	1	1	5	4	3
OTU_176	0	1	0	3	3	1	3
OTU_177	0	0	0	0	2	0	0
OTU_178	1	1	0	1	1	0	0
OTU_179	0	0	0	1	4	0	0
OTU_18	0	0	0	0	0	0	4
OTU_180	0	0	1	0	0	0	4
OTU_181	1	0	0	0	0	0	0
OTU_182	0	3	0	0	2	0	2
OTU_184	0	1	0	0	1	2	0
OTU_186	0	0	0	1	3	1	0
OTU_187	0	1	5	0	0	3	1
OTU_188	0	1	3	1	4	0	0
OTU_189	0	0	0	0	1	1	2
OTU_19	0	0	0	0	0	0	4
OTU_190	0	0	0	0	4	1	0
OTU_191	0	0	4	0	3	0	4
OTU_192	0	0	1	0	4	0	1
OTU_193	0	2	0	0	8	3	2

OTU_194	0	0	0	0	0	3	0
OTU_195	0	2	0	1	0	0	0
OTU_196	16	33	2	10	3	31	52
OTU_197	17	5	4	35	72	10	19
OTU_198	5	5	3	52	17	21	6
OTU_199	15	13	1	4	3	6	13
OTU_2	11	4	2	27	9	15	10
OTU_20	1	12	0	1	4	5	3
OTU_200	0	2	0	0	0	0	0
OTU_201	0	1	0	0	0	0	0
OTU_203	0	0	0	3	0	0	0
OTU_204	0	0	0	3	1	0	1
OTU_205	0	0	0	0	0	0	0
OTU_206	0	1	0	0	3	1	0
OTU_207	0	778	1	1	0	0	1
OTU_208	230	278	4	2	33	3	11
OTU_209	16	0	3	0	0	2	48
OTU_21	2	6	3	4	6	13	5
OTU_210	1	0	0	0	0	0	1
OTU_211	0	0	1	0	0	0	3
OTU_212	29	1	251	137	51	307	16
OTU_213	0	0	0	0	0	3	0
OTU_214	1	0	3	2	0	1	1
OTU_215	0	2	0	0	0	0	0
OTU_216	0	0	3	0	0	3	0
OTU_217	0	0	1	4	3	1	0
OTU_218	0	0	0	0	1	1	0
OTU_219	0	0	0	0	0	17	0
OTU_22	0	29	0	0	5	0	0
OTU_220	0	0	0	2	0	0	0
OTU_221	4	2	2	1	0	7	47
OTU_222	1	1	0	0	0	0	1
OTU_223	5	1	0	0	0	3	49
OTU_224	0	2	0	0	1	1	4
OTU_225	47	732	748	654	856	530	21
OTU_226	22	87	19	160	132	59	231
OTU_227	186	429	216	109	204	266	53
OTU_228	0	0	0	1	0	0	1
OTU_229	9	11	80	40	19	95	160
OTU_23	2	6	1	0	6	2	1
OTU_230	54	240	23	65	198	148	275
OTU_231	100	80	98	23	11	64	19
OTU_232	57	19	61	105	72	31	14
OTU_233	35	57	47	81	53	70	48
OTU_234	65	0	0	22	8	45	118
OTU_235	10	57	1	15	27	27	47
OTU_236	5	0	3	16	124	12	31
OTU_237	67	32	28	6	17	3	91
OTU_238	58	292	14	70	219	122	119
OTU_239	9	17	38	53	14	49	36
OTU_24	2	0	2	1	1	0	2

OTU_240	7	9	5	61	9	35	8
OTU_241	2	0	1	112	43	20	3
OTU_242	6	5	1	16	12	10	39
OTU_243	0	314	61	2	0	0	4
OTU_244	0	0	0	24	7	0	20
OTU_245	9	19	16	2	0	6	49
OTU_246	3	3	8	21	7	29	12
OTU_247	42	12	15	6	16	21	10
OTU_248	38	34	18	20	10	17	1
OTU_249	0	0	68	9	0	0	0
OTU_25	2	0	0	0	2	1	0
OTU_250	9	5	7	14	5	4	5
OTU_251	8	17	3	11	4	14	5
OTU_252	1	2	2	6	3	10	7
OTU_253	4	2	3	1	4	6	8
OTU_254	0	1	1	3	4	6	14
OTU_255	2	1	2	2	13	7	0
OTU_256	5	2	10	5	1	2	5
OTU_257	397	1041	0	104	937	664	346
OTU_258	2699	2	2577	1647	33	55	2450
OTU_259	12	2	6	2	0	1	1
OTU_26	3	0	0	6	1	1	0
OTU_260	1	0	0	6	2	5	4
OTU_261	2	0	0	2	0	0	8
OTU_262	0	0	16	6	0	0	0
OTU_263	0	0	0	0	0	0	10
OTU_264	0	0	0	4	4	1	23
OTU_265	0	0	0	0	0	0	0
OTU_266	1	2	1	0	0	7	0
OTU_267	0	0	0	2	0	0	0
OTU_268	0	1	0	1	0	2	4
OTU_269	27	41	56	0	36	43	32
OTU_27	0	0	0	0	0	0	3
OTU_270	162	0	556	2	0	147	107
OTU_271	3	7	36	5	54	15	16
OTU_272	4	0	57	32	39	36	6
OTU_273	58	0	33	61	97	339	37
OTU_274	1	6	0	46	14	29	48
OTU_275	4	97	48	15	643	7	6
OTU_276	0	8	0	5	3	0	10
OTU_277	0	26	0	6	2	4	14
OTU_278	0	7	1	0	0	0	0
OTU_279	0	0	0	1	0	0	0
OTU_28	1	0	0	4	4	0	2
OTU_280	1	0	0	0	0	1	5
OTU_281	2	19	54	6	10	39	16
OTU_282	1	12	1	20	215	22	52
OTU_283	0	0	0	0	0	0	1
OTU_284	2	0	1	0	0	3	2
OTU_285	6	32	6	39	31	26	190
OTU_286	125	1149	162	121	172	52	777

OTU_287	0	9	0	0	2	2	11
OTU_288	0	0	0	0	0	0	0
OTU_289	0	1	0	0	0	1	0
OTU_29	1	0	0	6	0	0	0
OTU_290	1	0	18	14	11	24	2
OTU_291	3	0	13	21	31	1	2
OTU_292	60	22	15	111	260	35	68
OTU_293	6	67	3	67	106	109	46
OTU_294	0	0	0	2	0	5	0
OTU_295	0	57	0	0	0	5	0
OTU_296	0	0	0	220	2	79	53
OTU_297	0	0	0	3	3	0	7
OTU_298	0	0	0	1	1	0	7
OTU_299	0	0	0	0	0	0	3
OTU_3	5	12	7	10	12	12	22
OTU_30	0	0	0	0	2	1	2
OTU_300	5	5	4	20	14	21	28
OTU_301	0	0	0	1	0	0	1
OTU_302	234	17	110	40	68	58	300
OTU_304	6	53	4	23	26	87	130
OTU_305	8	59	2	16	9	32	82
OTU_306	0	0	0	0	0	0	0
OTU_307	0	0	7	8	0	0	0
OTU_308	0	0	0	0	0	0	0
OTU_309	0	0	0	0	2	0	12
OTU_31	3	0	0	0	7	0	0
OTU_310	0	0	4	8	0	8	0
OTU_311	3	0	1	1	0	2	36
OTU_312	0	6	8	7	7	6	11
OTU_314	204	176	216	43	400	41	43
OTU_315	1	0	0	8	5	3	0
OTU_316	0	0	0	2	0	3	2
OTU_317	1	6	0	1	4	11	76
OTU_318	344	0	84	80	174	120	62
OTU_319	0	0	1	1	6	1	3
OTU_32	0	0	0	4	0	6	0
OTU_320	4	0	13	17	7	13	30
OTU_321	3	1	10	14	41	4	7
OTU_322	1	4	2	11	18	7	17
OTU_323	0	0	0	0	3	0	1
OTU_324	1	0	25	0	29	10	0
OTU_325	0	6	1	1	6	0	5
OTU_326	0	0	0	1	0	102	1
OTU_327	28	56	35	39	61	42	35
OTU_328	0	84	0	85	25	6	12
OTU_329	20	99	4	39	75	55	147
OTU_33	0	0	1	7	3	0	1
OTU_330	49	43	26	36	25	22	11
OTU_331	24	16	30	55	22	22	8
OTU_332	3	0	4	102	15	39	5
OTU_333	0	0	0	60	0	15	0

OTU_334	2	40	5	5	139	11	60
OTU_335	4	10	5	18	60	18	45
OTU_336	4	55	0	0	1	0	106
OTU_337	56	60	41	28	23	30	1
OTU_338	18	0	19	10	10	28	5
OTU_339	0	16	0	6	30	28	23
OTU_34	0	0	0	2	0	0	0
OTU_340	3	0	0	10	3	25	1
OTU_341	0	0	9	0	0	3	0
OTU_342	3	31	0	9	13	6	38
OTU_343	6	25	12	4	1	5	4
OTU_344	4	41	1	10	10	16	43
OTU_345	3	6	0	6	4	4	9
OTU_346	0	2	1	0	2	6	10
OTU_347	5	0	0	10	3	8	12
OTU_348	5	1	5	9	8	14	8
OTU_349	35	148	10	28	83	99	86
OTU_35	6	0	0	0	0	2	0
OTU_350	4	9	4	2	6	5	5
OTU_351	0	3	3	0	0	3	4
OTU_352	0	0	0	6	0	0	0
OTU_353	0	0	1	6	1	0	35
OTU_354	0	0	0	0	18	0	0
OTU_355	0	0	1	2	2	3	0
OTU_356	117	0	36	39	19	212	101
OTU_357	0	0	0	1	5	0	0
OTU_358	0	0	1	0	0	1	5
OTU_359	7	22	3	7	31	28	54
OTU_36	1	0	0	4	1	0	0
OTU_360	0	1	0	0	3	2	9
OTU_361	0	0	0	1	2	2	0
OTU_362	28	0	37	1034	151	281	2
OTU_363	5	2	3	11	28	1	2
OTU_364	0	2	0	1	9	0	33
OTU_365	0	0	1	2	18	11	2
OTU_366	0	9	1	8	2	0	27
OTU_367	0	0	0	1	0	4	15
OTU_368	3	1	4	0	1	3	10
OTU_369	0	0	0	0	0	1	12
OTU_37	5	3	4	0	0	0	0
OTU_370	36	110	27	24	14	19	45
OTU_371	3	0	0	0	0	5	0
OTU_372	1	29	0	0	0	8	3
OTU_373	0	0	0	0	1	0	25
OTU_374	0	0	4	4	3	0	10
OTU_375	1	0	0	1	0	1	15
OTU_376	2	3	0	10	44	28	110
OTU_377	1	0	9	6	3	0	14
OTU_378	0	0	0	1	2	0	0
OTU_379	1	9	1	4	19	4	7
OTU_38	8	2	2	10	4	1	4

OTU_380	0	0	0	0	2	1	9
OTU_381	1	0	3	1	6	0	5
OTU_382	879	109	123	392	659	130	42
OTU_383	3	0	64	39	35	106	14
OTU_384	0	0	0	0	0	0	1
OTU_385	0	0	0	0	0	0	2
OTU_386	0	1	6	2	3	0	11
OTU_387	0	2	0	1	1	1	8
OTU_389	2	16	7	21	10	8	27
OTU_39	0	0	1	0	0	6	0
OTU_390	11	26	90	14	72	13	12
OTU_391	0	0	0	0	0	0	6
OTU_392	28	3	17	6	19	13	63
OTU_394	2	15	1	10	3	11	6
OTU_396	0	0	2	0	0	0	1
OTU_397	20	60	9	43	31	19	73
OTU_398	17	32	4	8	163	3	32
OTU_399	1	1	0	1	2	1	0
OTU_4	3	1	1	1	1	0	3
OTU_40	2	1	1	0	0	0	0
OTU_400	0	0	0	0	0	0	0
OTU_401	0	4	4	0	9	0	0
OTU_402	20	23	17	6	33	12	1
OTU_403	0	0	4	0	1	0	0
OTU_404	147	114	99	101	164	118	1
OTU_405	84	6	16	47	20	66	20
OTU_406	42	13	9	94	30	73	37
OTU_407	75	239	12	55	96	22	59
OTU_408	58	0	6	2	1	77	42
OTU_409	24	49	35	11	96	63	20
OTU_41	1	0	0	2	0	2	0
OTU_410	1	35	8	33	13	21	39
OTU_411	27	57	54	35	31	21	9
OTU_412	4	61	1	9	9	19	46
OTU_413	26	52	15	14	41	29	9
OTU_414	42	21	35	12	12	16	23
OTU_415	21	2	7	27	16	4	5
OTU_416	7	15	2	25	56	37	41
OTU_417	0	0	1	0	0	34	49
OTU_418	25	18	17	18	32	69	6
OTU_419	15	32	4	34	51	40	43
OTU_42	1	5	0	0	0	0	0
OTU_420	11	10	42	25	54	29	17
OTU_421	6	2	12	25	13	12	16
OTU_422	13	3	2	20	24	7	0
OTU_423	12	1	62	155	93	10	107
OTU_424	6	153	2	0	23	3	4
OTU_425	0	0	0	0	0	28	0
OTU_426	6	14	28	8	13	13	9
OTU_427	2	0	11	9	7	23	0
OTU_428	4	0	9	23	2	3	0

OTU_429	5	16	10	10	10	6	3
OTU_43	2	0	1	0	0	0	0
OTU_430	12	13	1	4	0	3	2
OTU_431	0	4	1	4	12	12	0
OTU_432	24	9	14	0	3	2	2
OTU_433	16	3	18	25	16	5	5
OTU_434	0	23	0	0	2	25	0
OTU_435	43	24	138	40	28	38	4
OTU_436	1	2	11	1	2	6	5
OTU_437	0	16	53	31	10	4	21
OTU_438	0	1	0	3	2	0	2
OTU_439	20	0	2	5	0	0	0
OTU_44	0	1	2	3	0	0	0
OTU_440	1	21	0	3	2	5	8
OTU_441	232	107	589	70	296	100	34
OTU_442	3	0	0	7	1	3	0
OTU_443	2	0	0	11	0	0	0
OTU_444	3	212	0	0	3	1	0
OTU_445	0	0	0	0	0	15	0
OTU_446	0	768	14	12	9	16	18
OTU_447	229	7	577	295	206	368	57
OTU_448	0	0	5	37	1	128	7
OTU_449	175	2	1	508	113	339	324
OTU_45	0	1	2	0	0	0	0
OTU_450	60	31	39	38	12	18	33
OTU_451	7	2	1	0	0	0	0
OTU_452	1	23	0	5	11	5	31
OTU_453	4	0	0	9	1	0	98
OTU_454	4	0	1	0	0	3	0
OTU_455	0	0	0	1	2	3	0
OTU_456	2	5	0	3	3	5	4
OTU_457	0	0	0	4	2	0	0
OTU_458	0	1	2	2	2	6	0
OTU_459	0	0	0	1	1	4	0
OTU_460	72	91	187	382	492	56	78
OTU_461	1	0	0	0	0	0	3
OTU_462	1	0	0	0	0	1	1
OTU_463	0	0	2	5	0	1	1
OTU_464	77	0	495	23	293	138	13
OTU_465	6	104	6	16	5	1	101
OTU_466	1	68	9	27	27	22	42
OTU_467	1	1	0	1	0	1	5
OTU_468	263	0	211	426	188	0	33
OTU_469	2	27	0	1	13	13	12
OTU_47	0	0	0	0	0	2	0
OTU_470	1	0	0	0	0	0	0
OTU_471	116	251	663	57	5	290	115
OTU_472	0	0	0	2	1	0	0
OTU_473	0	0	0	4	2	6	2
OTU_474	16	67	35	71	84	42	16
OTU_475	17	5	14	1	26	2	1

OTU_476	0	3	0	0	0	0	2
OTU_477	137	87	0	39	19	124	294
OTU_478	1	4	0	0	0	0	12
OTU_479	0	0	0	0	0	0	4
OTU_48	3	0	0	0	0	0	0
OTU_480	0	0	0	0	2	1	5
OTU_481	0	0	0	0	0	0	1
OTU_482	0	0	0	0	0	0	2
OTU_483	1	0	6	9	5	16	21
OTU_484	1	0	3	0	0	2	0
OTU_485	15	24	9	47	41	23	74
OTU_486	4	6	0	1	47	5	32
OTU_488	4	23	9	15	33	5	19
OTU_489	2	3	0	4	9	1	7
OTU_49	0	3	0	0	0	0	0
OTU_490	0	0	1	9	2	168	0
OTU_491	0	1	0	0	1	1	0
OTU_492	0	0	0	0	0	14	0
OTU_493	0	3	1	0	2	1	0
OTU_494	0	0	0	2	9	4	3
OTU_495	0	0	0	343	0	89	0
OTU_496	16	97	8	86	144	47	91
OTU_497	0	3	12	14	26	7	0
OTU_498	0	0	0	2	7	0	0
OTU_499	0	0	3	25	26	6	0
OTU_5	3	0	2	0	0	0	1
OTU_50	2	21	5	255	52	30	0
OTU_500	6	54	2	61	5	2	3
OTU_501	8	0	0	74	1	1	1
OTU_502	16	29	2	169	286	70	17
OTU_503	0	0	0	0	0	0	0
OTU_504	0	0	0	1	0	0	0
OTU_505	0	0	0	0	0	0	0
OTU_506	64	219	325	286	43	250	9
OTU_507	0	0	0	1	4	0	0
OTU_508	0	0	1	2	2	2	0
OTU_509	0	1	1	1	2	1	0
OTU_51	26	27	9	16	27	42	40
OTU_510	73	63	204	68	46	182	40
OTU_511	2	11	7	14	9	10	12
OTU_512	0	1	0	0	2	0	0
OTU_513	1237	474	898	35	248	678	204
OTU_514	55	404	27	10	23	35	41
OTU_515	28	17	8	6	184	33	135
OTU_516	121	53	118	56	60	34	28
OTU_517	68	62	9	112	63	43	38
OTU_518	5	7	2	15	52	0	223
OTU_519	248	5	3	0	59	0	2
OTU_52	3	16	5	32	47	33	11
OTU_520	15	48	8	34	64	51	66
OTU_521	120	42	42	24	85	55	3

OTU_522	16	60	36	31	26	46	40
OTU_523	0	7	19	14	0	0	24
OTU_524	3	4	26	20	7	16	33
OTU_525	1	0	0	0	0	121	19
OTU_526	0	582	1	0	0	138	0
OTU_527	0	2	0	3	6	102	1
OTU_528	13	6	5	17	5	29	15
OTU_529	2	6	7	23	9	76	51
OTU_53	0	2	2	10	3	24	1
OTU_530	17	24	38	16	42	15	16
OTU_531	14	19	17	12	11	7	10
OTU_532	54	78	52	141	63	103	69
OTU_533	10	26	8	60	32	35	39
OTU_534	1	12	14	15	31	80	11
OTU_535	6	22	10	22	6	8	17
OTU_536	0	0	6	70	0	10	7
OTU_537	0	10	0	41	9	0	0
OTU_538	30	12	112	45	22	6	38
OTU_539	10	7	0	1	5	2	1
OTU_54	2	0	1	12	0	15	0
OTU_540	0	0	0	1	0	35	0
OTU_541	96	45	196	189	223	61	93
OTU_542	4	3	24	45	21	26	10
OTU_543	10	48	2	2	3	12	6
OTU_544	1	3	1	10	7	7	12
OTU_545	15	101	30	53	52	44	89
OTU_546	5	5	50	11	3	7	4
OTU_547	26	126	56	42	110	27	84
OTU_548	22	107	39	38	114	84	62
OTU_549	1	2	4	5	12	2	8
OTU_55	0	0	0	0	0	2	0
OTU_550	28	0	18	27	11	23	8
OTU_551	1	1	19	28	15	4	0
OTU_552	0	3	0	3	6	8	5
OTU_553	0	3	3	0	2	6	12
OTU_554	1	0	0	0	1	7	3
OTU_555	0	26	29	21	16	12	14
OTU_556	0	3	0	0	4	0	3
OTU_557	3	1	0	5	0	0	12
OTU_558	1	1	0	0	0	15	0
OTU_559	0	0	0	4	1	0	0
OTU_56	0	0	0	2	1	0	0
OTU_560	6	3	0	3	3	9	3
OTU_561	3	2	0	4	10	6	20
OTU_562	4	0	3	2	7	0	1
OTU_563	171	18	151	371	61	131	290
OTU_564	0	0	0	0	0	17	0
OTU_565	39	370	15	62	13	60	98
OTU_566	0	0	0	0	0	18	0
OTU_567	1	0	0	0	4	0	9
OTU_568	11	0	1	10	1	31	4

OTU_569	158	0	51	19	132	8	80
OTU_57	2	0	0	1	0	1	1
OTU_570	185	160	119	184	247	52	37
OTU_571	0	4	7	4	6	6	3
OTU_572	1	2	1	8	4	1	2
OTU_573	317	164	95	1	2	174	87
OTU_574	0	0	0	1	2	9	0
OTU_575	5	1	3	2	1	0	1
OTU_576	57	64	1	43	267	43	264
OTU_577	3	1	8	6	5	10	6
OTU_578	0	0	0	0	2	2	4
OTU_579	1	0	1	52	17	43	3
OTU_58	54	30	9	68	28	81	125
OTU_580	0	0	0	5	0	0	0
OTU_581	2	0	23	4	0	1	15
OTU_582	0	0	0	0	2	5	14
OTU_583	1	2	0	2	9	3	2
OTU_584	0	4	0	0	9	2	1
OTU_585	2	5	3	2	3	1	2
OTU_586	9	6	20	1	13	53	22
OTU_587	41	190	38	63	70	57	16
OTU_588	19	5	1	28	18	3	284
OTU_589	0	2	0	3	3	3	5
OTU_59	0	1	0	0	0	1	2
OTU_590	0	4	0	0	1	0	8
OTU_591	1	4	12	0	1	7	4
OTU_592	0	0	3	2	0	0	0
OTU_593	23	0	60	57	2	2	150
OTU_594	0	6	20	4	6	29	4
OTU_595	6	0	0	0	0	2	0
OTU_596	75	1	439	273	7	457	840
OTU_597	239	5	1420	645	265	1500	138
OTU_598	1	0	0	2	0	0	0
OTU_599	6	0	1	4	10	23	7
OTU_6	0	0	0	1	0	1	0
OTU_60	24	1	1	6	3	22	21
OTU_600	0	7	0	0	0	3	0
OTU_601	65	50	36	54	60	27	37
OTU_602	56	0	2	1	2	4	133
OTU_603	0	1	0	1	0	0	0
OTU_604	47	114	26	53	21	26	151
OTU_605	1	0	2	0	2	2	0
OTU_606	0	0	0	0	1	0	14
OTU_607	8	0	3	0	0	0	7
OTU_608	0	0	0	2	0	1	0
OTU_609	1	0	0	0	0	1	0
OTU_61	136	32	42	293	74	357	107
OTU_610	10	14	8	122	66	28	42
OTU_611	0	3	1	3	1	2	0
OTU_612	0	0	0	2	1	1	4
OTU_613	64	21	146	53	35	15	54

OTU_614	0	0	1	0	20	0	11
OTU_615	10	55	22	40	28	26	48
OTU_616	10	15	15	0	0	17	23
OTU_617	0	0	1	5	1	0	2
OTU_618	0	0	0	1	0	0	0
OTU_619	0	0	0	0	0	0	1
OTU_62	0	0	0	0	0	0	1
OTU_620	0	0	13	14	1	9	44
OTU_621	0	10	1	1	2	0	5
OTU_622	7	14	16	1	15	19	12
OTU_623	1	1	3	6	1	2	6
OTU_624	2	17	0	1	3	3	1
OTU_625	0	7	0	0	0	0	0
OTU_626	0	0	0	6	3	7	5
OTU_627	0	0	0	1	0	0	0
OTU_628	0	0	0	0	1	1	0
OTU_629	1	1	1	2	0	0	0
OTU_63	46	83	27	136	105	115	69
OTU_630	0	0	0	1	1	1	0
OTU_631	0	0	0	0	0	0	2
OTU_632	5	11	1	6	13	25	33
OTU_633	0	0	1	1	0	1	8
OTU_634	0	0	0	1	0	0	3
OTU_635	0	0	0	0	6	2	0
OTU_636	0	0	0	2	0	0	1
OTU_637	0	1	0	0	0	0	11
OTU_638	0	0	1	1	3	18	6
OTU_639	0	0	0	0	0	0	1
OTU_64	445	166	77	25	4	100	82
OTU_640	0	0	0	0	0	0	1
OTU_641	0	0	0	0	2	1	5
OTU_642	0	2	1	1	12	2	25
OTU_643	0	0	0	0	0	0	2
OTU_644	0	0	9	3	2	5	4
OTU_645	0	1	0	0	0	0	0
OTU_646	3	8	6	9	2	10	17
OTU_647	0	1	0	0	0	1	7
OTU_648	0	0	0	22	0	13	9
OTU_649	0	1	6	14	1	1	68
OTU_65	385	272	217	90	86	83	205
OTU_650	0	0	0	0	0	14	7
OTU_651	0	0	0	0	0	0	1
OTU_652	0	0	0	0	5	3	0
OTU_653	0	1	0	0	1	0	0
OTU_654	0	0	24	8	7	31	5
OTU_655	0	0	0	1	4	0	0
OTU_656	3	143	6	7	58	18	0
OTU_657	0	0	88	102	0	2	0
OTU_658	1	1	2	0	0	1	0
OTU_659	0	0	0	2	0	0	0
OTU_66	75	27	457	10	337	81	97

OTU_660	1	0	2	0	0	1	4
OTU_661	913	70	343	269	712	200	24
OTU_662	10	21	1	13	106	11	71
OTU_663	23	0	10	88	81	126	39
OTU_664	0	0	1	1	0	0	0
OTU_665	0	0	0	2	0	0	0
OTU_667	0	0	3	0	2	6	0
OTU_668	11	22	15	54	18	19	4
OTU_669	0	5	3	2	10	0	1
OTU_67	154	44	447	107	443	112	97
OTU_670	0	2	0	1	0	0	0
OTU_671	0	0	0	0	1	0	0
OTU_672	0	0	0	0	5	0	2
OTU_675	0	1	0	0	0	0	0
OTU_676	0	0	1	0	2	1	0
OTU_677	0	0	0	0	2	0	0
OTU_678	4	0	24	1	2	47	0
OTU_679	0	1	0	2	1	0	3
OTU_68	211	139	74	116	174	202	83
OTU_680	0	1	0	0	1	1	0
OTU_681	0	2	0	0	0	0	1
OTU_682	0	5	0	1	1	2	2
OTU_683	0	9	0	0	0	4	3
OTU_684	0	44	0	0	0	2	0
OTU_685	19	46	147	10	9	51	52
OTU_686	53	84	97	41	5	7	7
OTU_687	31	1	11	37	3	2	12
OTU_688	8	0	1	23	10	0	27
OTU_689	7	0	3	15	9	12	8
OTU_69	23	16	25	9	2	188	82
OTU_690	25	7	6	1	4	8	3
OTU_691	4	3	1	6	10	3	8
OTU_692	1	2	20	18	2	1	0
OTU_693	5	4	1	6	2	4	1
OTU_694	3	0	6	12	1	1	1
OTU_695	1	0	0	1	0	0	1
OTU_696	1	0	1	2	1	0	0
OTU_697	0	0	0	0	0	0	1
OTU_698	0	0	0	1	0	0	0
OTU_699	0	0	0	0	1	0	0
OTU_7	135	70	2	113	54	187	138
OTU_70	73	70	31	84	136	452	34
OTU_701	0	1	0	0	2	0	0
OTU_702	0	0	0	0	0	1	0
OTU_703	0	0	0	0	0	7	0
OTU_704	0	0	0	17	0	2	0
OTU_705	0	0	0	5	0	2	1
OTU_707	0	0	0	1	0	0	0
OTU_71	32	23	29	112	116	94	2
OTU_72	47	40	12	3	5	29	99
OTU_73	135	15	86	129	51	100	89

OTU_74	52	22	9	11	3	96	48
OTU_75	38	10	24	13	26	19	31
OTU_76	100	41	45	32	25	37	41
OTU_77	24	25	13	30	18	35	12
OTU_78	21	23	16	18	18	20	46
OTU_79	1	1	0	6	2	7	11
OTU_8	417	228	168	81	17	74	10
OTU_80	1	1	0	3	8	13	3
OTU_81	0	0	0	2	2	2	2
OTU_82	2	0	3	1	0	5	10
OTU_83	0	0	0	3	0	17	0
OTU_84	1	0	0	1	11	2	4
OTU_85	0	0	1	1	2	0	4
OTU_86	0	0	0	4	0	3	0
OTU_87	0	0	4	1	1	0	1
OTU_88	229	553	24	266	196	226	103
OTU_89	1	2	0	1	3	3	0
OTU_9	2	16	12	3	41	17	7
OTU_90	1	0	0	2	0	2	0
OTU_91	0	0	0	0	1	1	2
OTU_92	0	2	0	0	1	1	1
OTU_93	1	0	4	0	2	0	5
OTU_94	0	0	2	1	0	1	1
OTU_95	1	0	1	0	4	1	2
OTU_96	0	3	1	1	2	0	1
OTU_97	0	0	0	1	3	1	1
OTU_98	0	2	0	1	1	0	0
OTU_99	0	0	2	0	5	1	3

B28BP22	B28BP23
275	260
0	9
2	4
1	1
2	5
3	5
1	0
3	2
7	4
2	1
3	5
1	1
9	4
1	1
1	2
2	1
3	6
0	0
1	5
0	2
0	1
1	2
6	3
0	1
0	3
0	0
0	0
104	74
1	4
1	0
0	0
3	2
11	13
0	2
3	1
2	0
1	1
1	0
0	1
1	0
0	0
0	0
39	23
8	1
0	5
1	0
217	218
90	178
41	109
13	23

4	30
3	62
9	0
2	5
4	0
4	5
4	1
0	2
0	2
1	2
1	0
0	1
1	2
0	1
0	3
0	0
2	6
1	3
6	1
0	1
2	4
2	1
0	0
0	1
5	4
1	0
6	2
2	2
0	2
0	1
3	5
4	0
1	1
6	5
1	1
2	0
0	6
13	12
0	1
0	0
0	0
1	2
0	2
2	3
0	0
1	1
5	4
0	0
1	2
0	1
2	1

0	2
0	1
31	97
18	1
16	9
0	1
10	8
1	4
0	1
0	2
0	0
0	1
0	2
0	1
0	0
6	38
81	18
0	8
1	0
2	0
130	91
5	1
0	1
0	11
2	0
1	0
0	0
0	0
0	4
0	0
2	15
0	0
0	2
5	1
471	163
224	93
193	52
1	0
84	95
1	0
253	166
56	73
32	25
50	74
68	1
65	34
95	51
21	95
110	61
60	36
1	1

28	0
17	11
14	48
0	0
75	0
0	22
4	18
0	0
1	16
2	0
0	3
6	6
7	8
8	1
9	3
7	23
3	0
4	7
1377	680
220	1178
0	2
0	1
0	0
0	0
3	0
0	0
8	4
0	68
0	1
1	0
0	0
70	41
9	4
876	0
33	25
50	47
87	22
27	13
3	46
2	5
13	4
0	36
4	0
0	0
0	0
7	20
44	64
1	0
0	3
42	77
160	501

9	1
1	0
0	3
1	0
18	10
6	1
50	1
56	49
0	0
0	0
139	0
1	3
7	2
0	1
11	4
2	0
6	1
0	1
80	149
29	44
26	52
2	0
7	5
286	35
15	22
0	0
3	1
67	11
31	13
112	55
0	2
0	0
24	45
28	259
3	1
0	1
13	9
4	11
10	10
0	1
28	2
2	1
1	0
15	12
0	51
187	141
3	0
13	19
26	36
4	16
10	4

10	47
49	64
0	0
6	12
10	5
19	42
2	0
16	0
65	2
10	42
3	12
23	34
6	7
0	13
7	16
8	16
65	144
0	0
8	6
1	3
12	10
2	2
0	0
1	1
61	30
4	4
3	1
24	40
0	1
7	6
0	0
18	94
2	5
2	0
16	22
9	3
5	4
1	17
0	2
0	0
30	11
0	0
5	1
1	0
8	2
0	6
3	36
8	2
0	2
2	4
1	0

2	2
0	21
48	123
44	45
0	0
0	3
2	5
8	7
7	22
2	0
18	10
0	6
10	21
9	6
1	1
32	31
109	16
1	1
8	2
0	0
1	0
0	4
12	4
0	0
114	49
21	41
23	23
60	43
16	38
5	2
1	6
11	4
20	16
53	48
10	11
10	9
1	2
50	22
1	2
11	31
39	69
0	0
14	12
15	2
4	0
93	25
1	35
0	161
6	6
13	58
9	4

7	17
0	0
4	1
0	1
2	3
27	3
0	2
14	23
7	7
14	7
6	4
0	0
0	0
4	3
235	184
1	6
0	0
8	2
0	13
31	18
249	271
64	14
121	86
0	1
24	18
0	2
10	8
174	236
0	0
1	1
2	0
5	3
0	3
1	5
302	323
0	0
0	1
1	0
163	6
18	70
23	36
0	3
78	13
26	18
0	3
0	0
62	68
0	0
0	1
10	21
1	1

0	0
114	190
5	3
0	2
0	0
0	1
0	0
0	0
8	6
0	0
41	63
13	13
13	27
14	4
0	0
486	5
3	1
276	5
3	6
15	7
90	0
40	123
6	4
1	0
3	3
0	0
18	3
33	30
2	0
46	33
1	0
0	0
0	1
0	79
0	0
0	0
1	0
48	16
45	8
10	11
0	1
571	388
51	31
69	47
28	22
8	23
46	1
2	0
3	19
16	29
17	23

66	43
102	2
21	30
3	265
0	3
2	0
21	23
47	65
7	34
13	18
8	17
108	102
60	45
31	36
23	16
0	4
8	0
49	16
25	3
6	0
0	203
64	54
15	8
12	1
1	5
85	65
1	5
65	82
46	24
17	3
0	0
19	51
1	14
2	5
6	8
11	24
19	4
35	2
2	13
1	7
13	94
0	0
3	11
18	5
3	5
139	120
0	0
83	91
0	9
1	0
8	1

47	378
1	1
75	37
1	3
5	1
1	336
10	7
0	0
157	118
13	3
3	2
47	12
120	243
0	1
0	7
6	0
2	7
3	4
2	2
18	24
29	28
21	34
15	5
3	3
1	3
5	4
0	1
17	84
7	3
0	1
324	428
676	493
2	0
11	2
0	3
14	1
0	5
39	29
11	1
1	4
157	34
0	1
0	0
0	0
2	1
1	0
202	252
211	122
3	0
3	8
56	25

5	37
20	17
0	48
0	4
0	0
3	1
0	0
5	9
3	5
27	28
1	2
5	11
2	0
7	11
0	0
0	0
0	0
139	259
1	1
0	0
9	12
0	7
0	0
0	0
1	1
0	0
5	1
0	1
76	77
0	0
1	3
10	6
1	1
8	6
0	0
12	12
0	1
12	1
18	31
215	366
0	28
2	0
4	0
1	0
32	20
1	1
156	62
251	62
1	4
1	0
101	415

8	0
122	247
58	41
99	174
0	0
0	0
0	1
24	8
3	1
81	123
0	1
0	2
1	0
0	0
1	0
0	0
11	14
1	0
84	50
0	0
0	0
0	2
1	0
0	0
22	68
6	5
8	6
0	5
6	7
67	99
0	0
6	11
2	5
2	2
2	2
0	3
0	0
0	0
0	1
0	0
39	79
108	106
0	0
0	0
0	0
1	0
1	0
0	0
25	63
28	57
120	140

61	98
13	37
40	77
31	34
15	54
8	11
40	29
6	8
5	3
0	2
1	0
15	2
2	3
3	0
1	8
194	176
1	2
6	5
3	5
2	4
2	2
3	7
1	5
2	3
3	5
1	8
2	5
1	8
